# State Registration and Its Values

**Published:** 1920-10-02

**Authors:** 


### THE MATRONS' AND SISTERS' DEPARTMENT.

### STATE REGISTRATION AND ITS VALUES.

### VI. Can Registration be made Universal?

WE have endeavoured to show that a State register which includes the names of only part of the profession will be self-confessed a failure. Unless there be a consensus of opinion among trained nurses that registration is desirable and (being in existence) actually necessary, it will bring disunion and perhaps even disaster in its train. We have attempted before to explain how this may come about, but it is a matter of so much importance that it will bear repetition.

Let it be supposed that registration is accepted by only some of the fully-qualified women embraced under its provisions. It is certain that a large number of imperfectly trained nurses will immediately apply to be received under the *bona fide* clause within the fold. Should they outnumber the fully-trained and certificated nurses irretrievable harm will be done, for the public will become speedily aware that registration does not "count." The effects of aloofness on the part of nurses who may believe their employment so secure that they cannot be affected by measures designed for the profession at large will in that case be disastrous. The main body of nurses will drift along as before. Registered nurses will become a mere clique, and the register which should have united will divide for at least another generation. This process has been witnessed in many American states in which State registration fails to draw within its magic circle the women who ought to pull shoulder to shoulder.

Who then shall register, and how best can they be drawn to do so? It is to the training-schools that the registration authorities must look to make registration a reality. It is they alone who can induce nurses automatically on certification to apply for registration. There is no other possible way of bringing the profession into line, and this alone would in course of time achieve the unity so much desired of all. So much for the future.

But what of the nurses scattered all over the kingdom, all over the Empire, whom it is desired to gather together under one all-embracing scheme? Here again it is the training-schools which can best help. They are in touch each one with a long chain of alumni responsive to their call, and through their associations and leagues can make their influence felt far and wide. Do we not all know that in matters relating to their profession it is to the matron or sister under whom nurses were trained that they will turn when doubtful what to do? If this influence be exerted in the cause of State registration it will succeed. The matron's quiet remark "I would," outweighs a ton of talk from anyone else.

The training-schools cannot reach the entire body of practising nurses in institutions, in district work, in private practice. But the nursing profession is no longer a scattered agglomerate of rather helpless units. It has an organisation of its own. It has meeting grounds in many different centres. It has at last a voice. In this hour preceding registration it looks to the College of Nursing for a lead as regards State registration, and it will surely not look in vain. One of the main purposes for which the College was founded was to obtain State recognition for its members and for nurses in general. That boon has been secured, but it still remains to bring nurses as a body to accept it.

We cannot disguise from ourselves that this is the crux of the matter. The problem is to impress upon an enormous body of women, notoriously ignorant of the things which belong to their welfare, the duty of taking advantage without delay of the new privilege which lies within their reach. To do this requires a real campaign. If every member of the profession worthy to come in is to be brought in, then every one must begin to work at once and must go on working. There must be State registration meetings just as there used to be revival meetings. All nurses who are able to speak and persuade their fellows must speak on this subject, and those who have not yet learned to speak must study to acquire the art. There must be no institution in which the benefits of State registration are not systematically pointed out to the staff. There must be no nursing home, no institute for nurses, where the matter is not canvassed. Thus and thus only can public opinion be formed. Thus and thus only can the main body of nurses, too, too sluggish in looking after their own interests, be brought into one homogeneous body.

Let the dangers of a divided profession which have been experienced for many years be kept well in mind. Every nurse who remains outside the register is a potential danger. In the aggregate these unregistered, uninspected, uncontrolled nurses will have power to neutralise many of the benefits which their fellows expect to receive from the act of registration. Unless they are reduced to a disreputable minimum State registration will fail.

